# P-101. A Phase 3 International, Multi-Center, Randomized, Open-Label, Assessor-Blind Study to Evaluate the Efficacy and Safety of Minocycline/EDTA/Ethanol (Mino-Lok) Therapy (MLT) vs Site-Specific Antimicrobial Lock in Combination with Systemic Antibiotics in the Treatment of Catheter-Related or Central Line-Associated Bloodstream Infection

**DOI:** 10.1093/ofid/ofaf695.330

**Published:** 2026-01-11

**Authors:** Anne-Marie Chaftari, Vinay Rathore, Ray Y Hachem, Toan T Huynh, Paul P Cook, Onix Cantres-Fonseca, Mayur Ramesh, Mark E Rupp, Leonard Mermel, Myron S Czuczman, Alan Lader, Issam I Raad

**Affiliations:** MD Anderson UT, Houston, Texas; All India Institute of Medical Sciences, Raipur, Chhattisgarh, India; MD Anderson UT, Houston, Texas; Surgical Critical Care and Acute Care Surgery, Atrium Health, Charlotte, North Carolina; Brody School of Medicine at East Carolina University, Greenville, NC; Advent Health Transplant Institute Orlando, Florida; VA Carribean Health-system, San Juan,PR, Orlando, Florida; Henry Ford Hospital, Detroit, MI; University of Nebraska Medical Center, Omaha, NE; Warren Alpert Medical School of Brown University and Rhode Island Hospital, Providence, Rhode Island; Citius Pharmaceuticals, Cranford, New Jersey; Citius Pharmaceuticals, Inc., Cranford, New Jersey; MD Anderson UT, Houston, Texas

## Abstract

**Background:**

Catheter-related or central line-associated bloodstream infection (CRBSI/CLABSI) causes substantial morbidity and mortality. Managing CRBSI/CLABSI often involves removing the infected central venous catheter (CVC) and inserting a new one at a different vascular site. Currently, no adjunct antimicrobial lock therapy (in combination with systemic antibiotics) has been FDA-approved and is urgently needed. Our study evaluated a novel triple combination antimicrobial therapy (Mino Lok (MLT)) containing minocycline, EDTA, and ethanol. MLT has shown broad-spectrum in-vitro activity and positive results in a Phase 2 trial.

**Figure:**
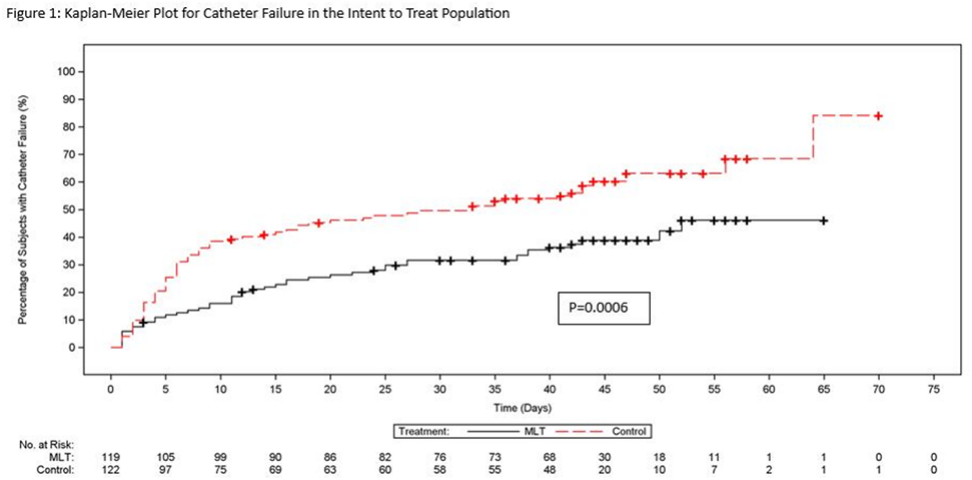

**Figure:**
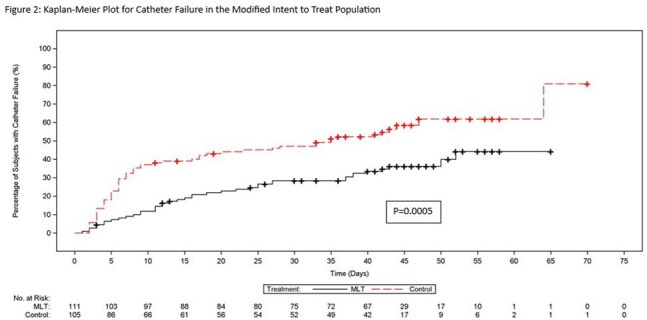

**Methods:**

This international, multicenter, superiority trial was conducted at 34 sites. Cancer, hemodialysis (HD), or other patients requiring a long-term CVC (LTCVC), aged ≥ 12 years, with CLABSI/CRBSI, were enrolled and randomized in a 1:1 ratio to receive either MLT or site-specific standard of care (SOC) antimicrobial lock for 2 hours/day for 7 days. The primary endpoint was median time to catheter failure (defined as mortality, catheter removal due to inability to administer lock or infectious-related reasons, worsening signs/symptoms, persistent or recurrent bloodstream infection, or deep-seated infection).

**Figure:**
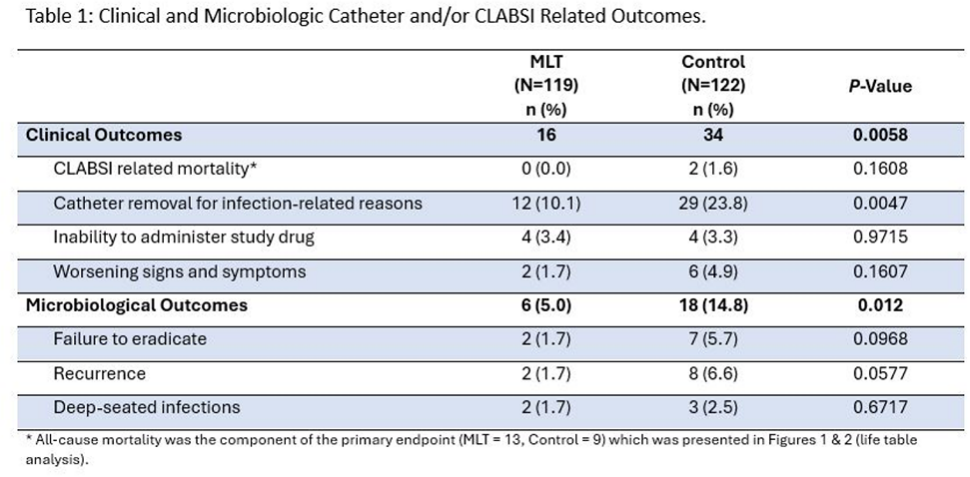

**Results:**

From February 2018 to February 2024, 241 subjects were enrolled and randomized with 228 receiving study drug. A significant difference in median time to catheter failure in intent-to-treat (ITT) and modified ITT (MITT) populations (p≤ 0.0006) was observed (Figures 1 & 2). The CVC was successfully retained in 57% of patients in MLT vs 38% in SOC (p=0.0025). Clinical and microbiological failure related to the catheter or CLABSI were significantly higher in SOC (p=0.0058 and p=0.012, respectively) (Table 1). Adverse events (AEs), serious AEs (SAEs) and all-cause mortality were comparable for the two groups. There were no drug-related SAEs.

**Conclusion:**

This phase 3 pivotal study demonstrated MLT to be highly effective and superior to SOC antimicrobial locks in salvaging LTCVCs associated with CRBSI/CLABSI in cancer, HD and other patients requiring LTCVC. MLT has broad-spectrum activity, was well-tolerated, and was not associated with drug-related SAEs. MLT may satisfy an urgent unmet need in the management of CRBSIs/CLABSI.

**Disclosures:**

Anne-Marie Chaftari, MD, Citius Pharmaceuticals, Inc., Cranford, New Jersey, USA: Grant/Research Support Vinay Rathore, MD, Citius Pharmaceuticals, Inc. 11 Commerce Drive, First Floor Cranford: Grant/Research Support Paul P. Cook, MD, Gilead: Grant/Research Support|Janssen: Grant/Research Support|Pfizer: Grant/Research Support Onix Cantres-Fonseca, MD, Citius Pharmaceuticals, Inc.: Grant/Research Support Mayur Ramesh, MD, Citius Pharmaceuticals, Inc.: Grant/Research Support Mark E. Rupp, MD, Armata: Advisor/Consultant|Citius Pharmaceuticals, Inc.: Advisor/Consultant|Magnolia: Grant/Research Support|Teleflex: Advisor/Consultant Leonard Mermel, DO, Citius Pharma: Advisor/Consultant|CorMedix Pharma: Advisor/Consultant|Destiny Pharma: Board Member|Lightline Medical: Advisor/Consultant|Pristine Access Technology: Advisor/Consultant|Pristine Access Technology: Stocks/Bonds (Private Company) Alan Lader, PhD, Citius Pharmaceuticals, Inc.: Senior Vice-President/employee Issam I. Raad, Distinguished Professor, Citius Pharmaceuticals, Inc. (Grant/Research Support): Advisor/Consultant|Citius Pharmaceuticals, Inc. (Grant/Research Support): Grant/Research Support|Citius Pharmaceuticals, Inc. (Grant/Research Support): Patent|Citius Pharmaceuticals, Inc. (Grant/Research Support): Ownership Interest|Citius Pharmaceuticals, Inc. (Grant/Research Support): Stocks/Bonds (Public Company)|Spectrum Vascular: Patent|Spectrum Vascular: Ownership Interest

